# Extrahepatic Mucinous Biliary Cystadenoma: A Case Report

**DOI:** 10.7759/cureus.10581

**Published:** 2020-09-22

**Authors:** Bandar R Bakhurji, Shymaa A Basager, Amani M Hakami, Manar A Bamashmoos, Manal H Alshammari

**Affiliations:** 1 Family and Community Medicine, Imam Abdulrahman Bin Faisal University, Dammam, SAU

**Keywords:** obstructive jaundice, biliary cystadenoma

## Abstract

Biliary cysts refer to cystic dilatation in the biliary ductal system that may be congenital or acquired. Extrahepatic biliary cysts constitute less than 10% of biliary cysts. Extrahepatic mucinous cystadenoma represents an extremely rare clinical condition with less than 100 cases reported in the English medical literature. Herein, we report the case of a middle-aged woman who presented with a clinical picture of cholestatic jaundice. Laboratory findings revealed elevated bilirubin and alkaline phosphatase. After a thorough investigation, she was found to have a cystic lesion in the common bile duct near the cystic duction site. The patient underwent exploratory laparotomy, which revealed a 2.0 × 2.0 cm cystic lesion in the common bile duct that is exerting an obstructive effect on the biliary ducts. Complete en-block excision of the cystic lesion was performed with Roux-en-Y hepaticojejunostomy reconstruction. Histopathological examination revealed mucinous biliary cystadenoma. Although very rare, biliary cystadenoma should be kept in mind as a differential diagnosis of cholestatic jaundice particularly in patients with no history of biliary stones or cholecystectomy.

## Introduction

Biliary cysts refer to cystic dilatation in the biliary ductal system that may be congenital or acquired. The cysts may be congenital or acquired. Extrahepatic biliary cysts constitute less than 10% of biliary cysts. Mucinous biliary cystadenoma represents an extremely rare clinical condition with less than 100 cases reported in the English medical literature [1]. Herein, we describe the case of a middle-aged woman who presented with cholestatic jaundice and was found to have biliary cystadenoma after thorough investigation.

## Case presentation

We report the case of a 48-year-old female patient who presented with a one-week history of worsening yellowish discoloration of the skin that was associated with dark urine, pale stool, and generalized itching. There was no history of nausea, vomiting, abdominal pain, or fever. Her past surgical history was significant for emergency laparoscopic cholecystectomy that she underwent six months ago at another institution for acute cholecystitis. The course of the operation was unremarkable for any complication. The patient did not smoke or drink alcohol and there was no family history of liver diseases.

**Figure 1:**
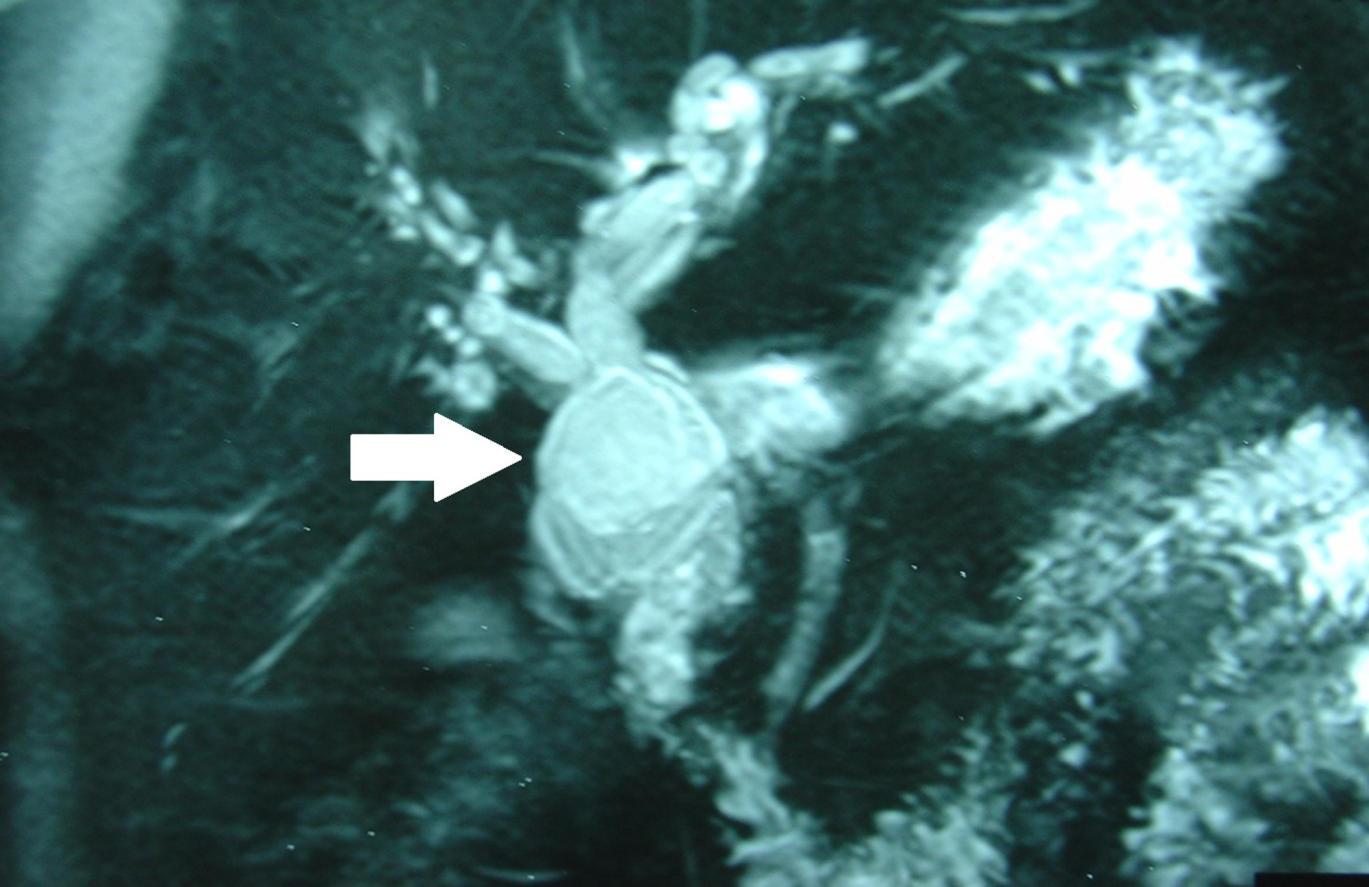
MRCP Image Magnetic resonance cholangiopancreatography showing a cystic lesion (arrow) in the common bile duct

Upon examination, the patient was icteric but afebrile and her blood pressure, pulse rate, and respiratory rate were observed to be 124/70 mm Hg, 88 beats per minute, and 18 breaths per minute, respectively. The abdominal examination revealed a soft and non-tender abdomen. Laboratory investigation showed elevated bilirubin (12.5 mg/dL), alkaline phosphatase (1450 IU/L), and the levels of other biochemical and hematological parameters were normal. In light of the laboratory findings, the patient underwent abdominal ultrasound, which demonstrated a dilated common bile duct of 13 mm in diameter with the presence of a cystic lesion measuring approximately 2.0 × 2.0 cm in maximum dimensions. A computed tomography (CT) of the abdomen was obtained to further characterize the lesion, which revealed a hypodense uniloculated cystic lesion between the porta hepatis and the pancreatic head. Subsequently, a magnetic resonance cholangiopancreatography (MRCP) revealed the thin-walled septated mass with significant intrahepatic biliary dilatation (Figure 1).

**Figure 2:**
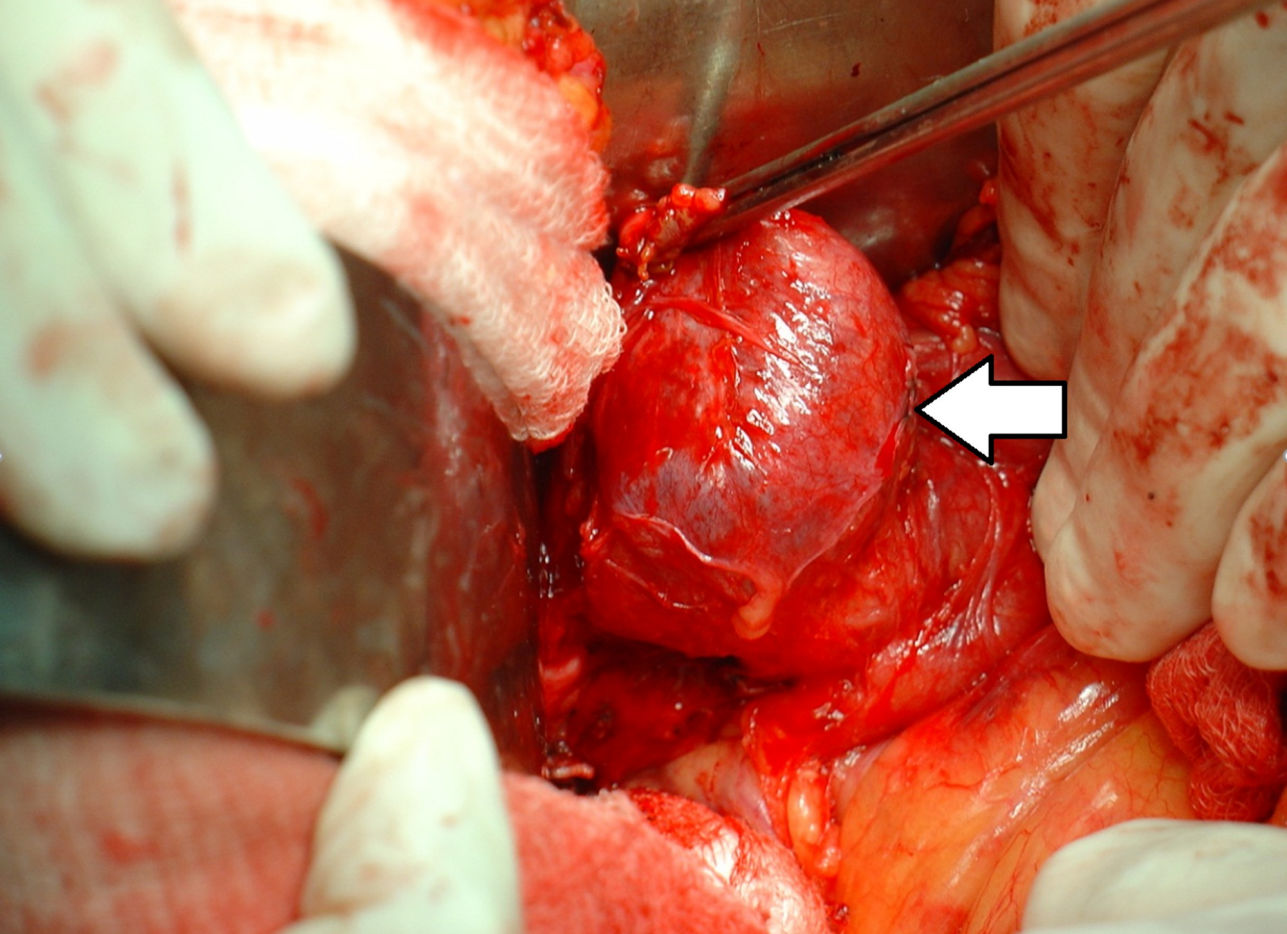
Operative Finding Laparotomy view demonstrating the cystic lesion (arrow) in the common bile duct

The radiological findings were discussed with the gastroenterology team who decided to proceed with endoscopic retrograde cholangiopancreatography (ERCP). No stones were retrieved and a nasobiliary drain was inserted. The patient was prepared for emergency diagnostic laparotomy. The exploration revealed a cystic dilatation in the common bile duct with gross dilatation proximally (Figure 2). The dilatation was at the site of the cystic duct stump.

**Figure 3:**
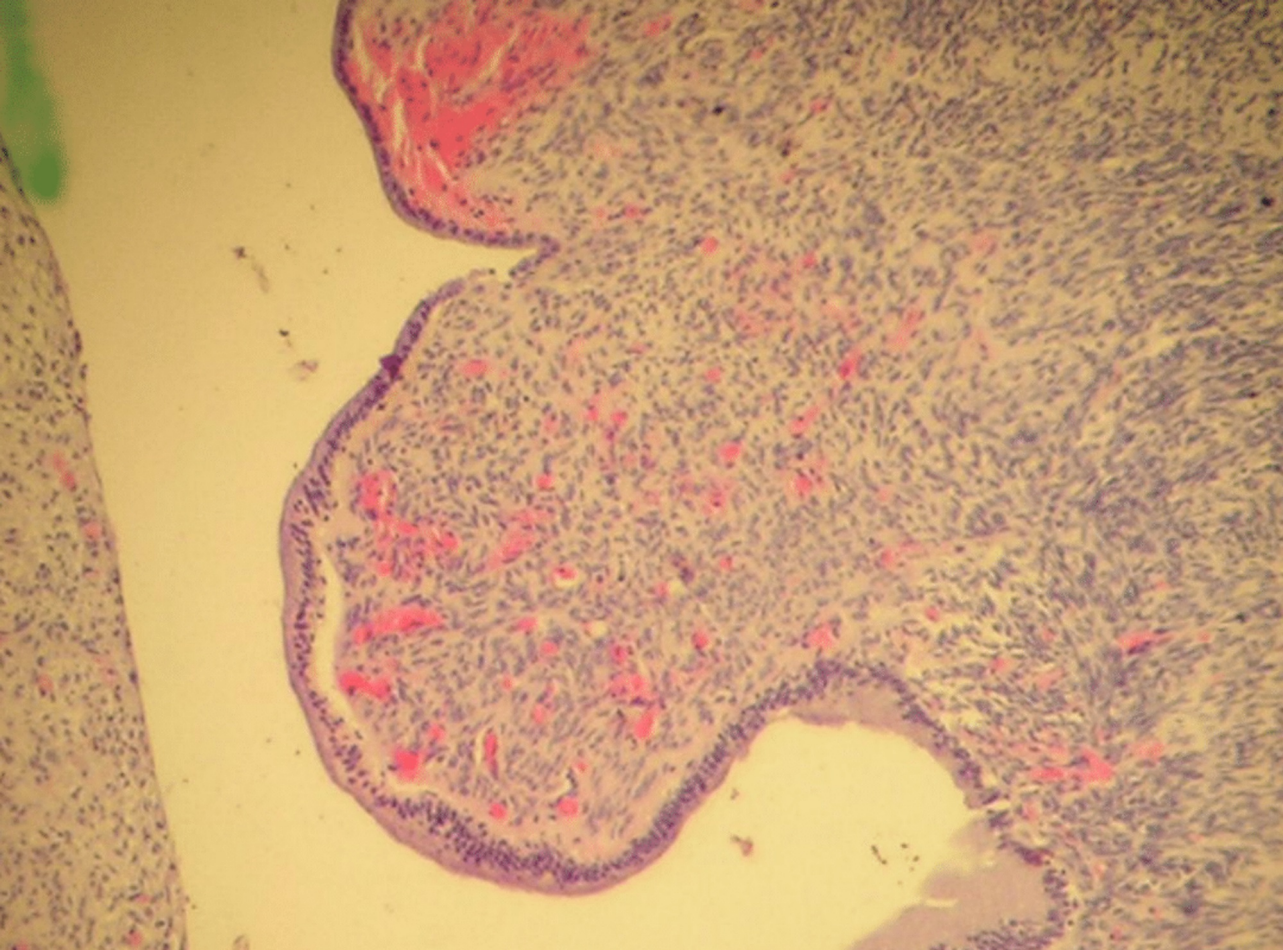
Histopathology Image (Low Power) Histopathological image showing the nodularity within the lesion

**Figure 4:**
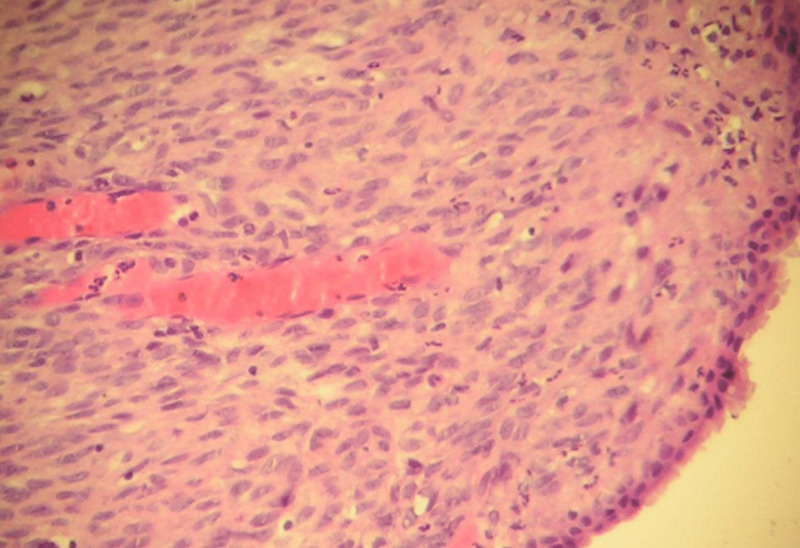
Histopathology Image (Medium Power) Histopathological image showing highly cellular ovarian-like stroma

**Figure 5:**
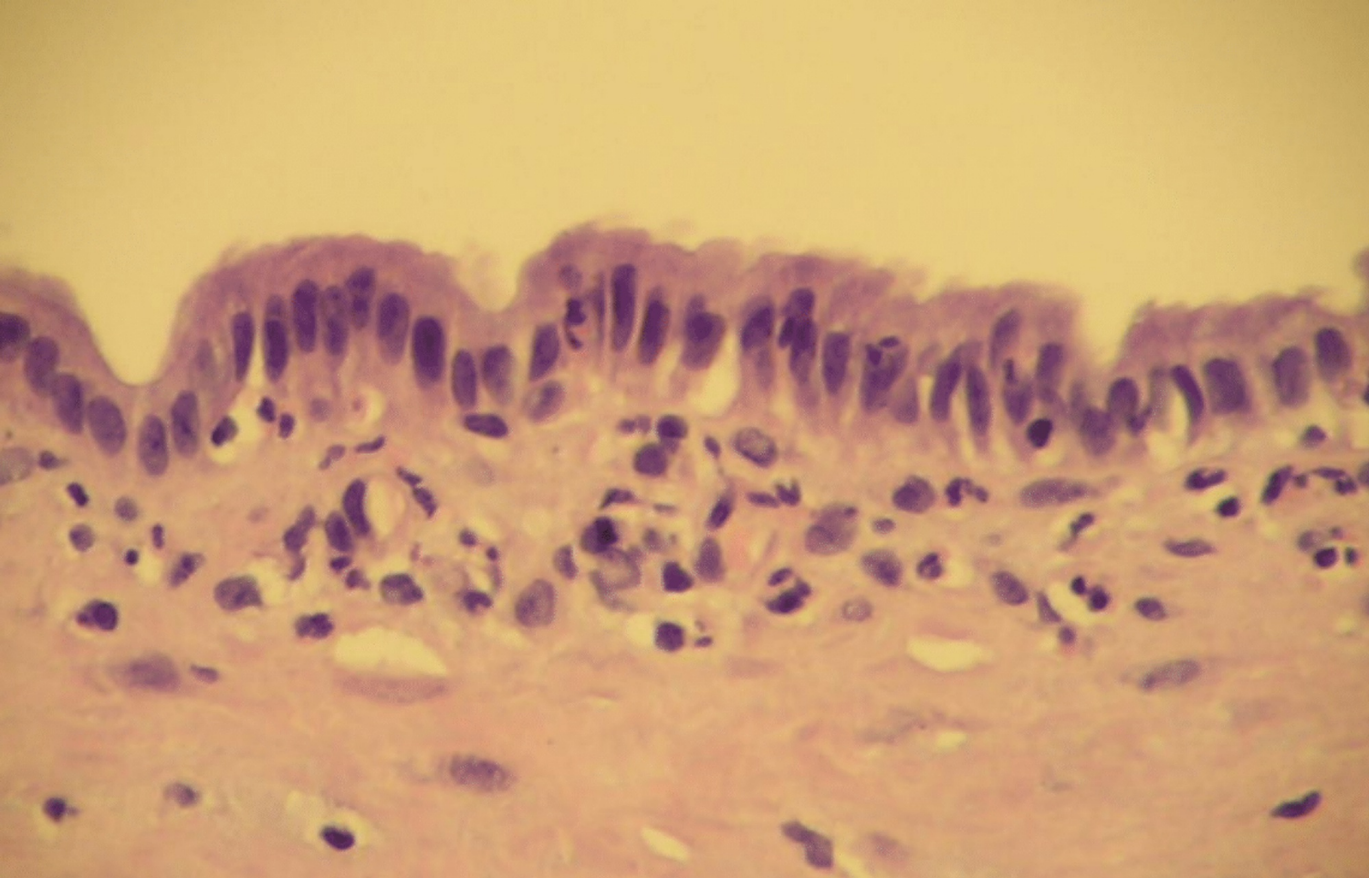
Histopathology Image (High Power) Histopathological image showing simple columnar biliary-type epithelium

Complete en-block excision of the cyst was performed with Roux-en-Y hepaticojejunostomy reconstruction. Gross examination of the resected cyst had a normal-appearing wall with mucoid material. Microscopic examination revealed the diagnosis of extrahepatic mucinous biliary cystadenoma (Figures 3-5).

## Discussion

We presented a case of extrahepatic mucinous cystadenoma presenting with cholestatic jaundice. This tumor typically occurs in middle-aged women and often poses a diagnostic challenge due to its rarity [2].

The pathogenesis of this condition remains unclear. It is suggested that its origin could be from congenital hamartomatous bile ducts [3]. However, some researchers believe that it may have a true neoplastic origin. Considering the female predominance of this tumor, hormonal factors could play a role in the pathogenesis. A prior study demonstrated estrogen and progestogen receptors in the cells of this tumor [4]. However, no immunohistochemical staining was performed in our case. Alternative theories suggest that it results from a reactive process to an acquired biliary injury [5].

Most cases of biliary cystadenomas are asymptomatic and diagnosed incidentally. Extrahepatic mucinous biliary cystadenoma may present with cholestatic jaundice, as in our case, and it has an earlier presentation as compared to its intra-hepatic counterpart. Other symptoms include vague abdominal pain, nausea/vomiting, anorexia, and weight loss [6].

None of the imaging modalities, including ultrasonography, CT, MRCP, and ERCP, have pathognomonic diagnostic features for extrahepatic mucinous biliary cystadenomas [7]. However, abdominal ultrasonography has a sensitivity of 90% in the detection of the lesion. The findings include an anechoic lesion with a thickened and irregular wall [8]. Additionally, mucinous biliary cystadenoma may be differentiated from a solitary bile duct cyst with high accuracy by using specific CT criteria [9]. However, the ability to consistently distinguish these lesions using imaging alone is controversial [10]. The differential diagnosis for biliary cystadenoma is very broad. It includes a wide range of conditions such as developmental, inflammatory, neoplastic, and other etiologies.

The malignant transformation of biliary cystadenoma may reach up to 20%. This risk is equal between males and females [11]. There are no treatment guidelines available for the management of extrahepatic biliary cystadenoma. However, considering the possibility of recurrence and malignant transformation, complete excision with negative margins followed by hepaticojejunostomy reconstruction is recommended [7].

## Conclusions

Although very rare, biliary cystadenoma should be kept in mind as a differential diagnosis of cholestatic jaundice, particularly in patients with no history of biliary stones or a cholecystectomy. Histopathological diagnosis is needed to establish the diagnosis. The case also shows the importance of discussing the case by the treating surgeon and the radiologist preoperatively.
